# The Origins of Intergroup Resource Inequality Influence Children’s Decision to Perpetuate or Rectify Inequality

**DOI:** 10.3389/fpsyg.2020.571570

**Published:** 2020-11-27

**Authors:** Jing An, Jing Yu, Liqi Zhu

**Affiliations:** ^1^CAS Key Laboratory of Behavioral Science, Institute of Psychology, Chinese Academy of Sciences, Beijing, China; ^2^Department of Psychology, University of Chinese Academy of Sciences, Beijing, China; ^3^Eunice Kennedy Shriver National Institute of Child Health and Human Development, Bethesda, MD, United States

**Keywords:** fairness, intergroup, resource inequality, structural origin, internal origin

## Abstract

Previous studies have explored children’s intergroup resource allocation in the context of preexisting intergroup resource inequality. However, resource inequality between social groups often originates from different factors. This study explored the role of the origins of resource inequality on children’s intergroup resource allocations. In experiment 1, when there was no explicit origin of the intergroup inequality, children of different ages mainly allocated resources in an equal way and 5- to 6-year-olds showed ingroup bias. In experiment 2, we examined the influence of different origins of intergroup inequality and found that 5- to 6-year-olds perpetuated intergroup inequality when resource inequality was based on either a structural (regional disparity) or an internal factor (difference in performance). However, 10- to 11-year-olds rectified inequality or allocated equally when intergroup inequality was based on regional disparity and perpetuated resource inequality when intergroup inequality was based on performance difference. The origins of inequality appear to play an important role in children’s intergroup resource allocations, and older children can distinguish different origins of intergroup inequality in resource allocation.

## Introduction

Inequalities are widespread in modern society, including economic, educational, and medical inequalities based on country, region, gender, race, or social class (Mara, 2014; Ridgeway, 2014; Normile, 2017). Understanding children’s recognition of inequality is of great significance for understanding the development of fairness considerations in social behavior and identifying ways to promote social equality. Resource inequality is an important form of social inequality (Ridgeway, 2014). In the face of resource inequality, how do children react and allocate resources?

Fairness is the criterion for resource allocation (Rutland and Killen, 2017), and the sense of fairness develops in early childhood. Infants expect resources to be allocated equally from the age of 10 months (Sloane et al., 2012; Meristo and Surian, 2013). When facing resource inequality between individuals, children aged 4–5 years old allocate more to individuals with fewer resources to reduce inequality (Li et al., 2014; Rizzo and Killen, 2016).

However, the unequal allocation of resources may occur due to many factors in the process of resource allocation, such as self-interest (Blake et al., 2014), performance and effort (Noh et al., 2019), and group affiliation (Yu et al., 2016). According to the impartiality account of fairness, inequality is not necessarily unfair (Shaw, 2016). Inequalities based on widely recognized factors, such as internal individual factors (e.g., performance, need, and effort) or random procedures, are considered to be fair, while inequalities based on self-interest, group membership, or structural factors (e.g., gender and race) are regarded as unfair (Shaw, 2016; Rizzo et al., 2020).

In early childhood, children begin to distinguish resource inequality between individuals based on these fair and unfair factors. Compared to equal allocation or higher allocation to ones with less labor, infants anticipate that two objects will receive unequal resources according to their merits (Sloane et al., 2012; Wang and Henderson, 2018). Children aged 3–8 years old perpetuate inequality based on performance differences, while they rectify resource inequality based on gender in resource allocation (Rizzo et al., 2020). When faced with resource inequality between themselves and others, 8-year-old children reject disadvantageous or advantageous inequality (Blake and McAuliffe, 2011; Shaw and Olson, 2012), but children accept inequality if disadvantageous inequality is determined by themselves (Shaw et al., 2016). Children also accept disadvantageous inequality in allocation when they are asked for opinions or when a fair procedure is used during the decision-making process (Grocke et al., 2015, 2018). These studies show that children can accept unequal outcomes with impartial procedures or reasonable justifications but reject inequality based on partial or unreasonable reasons.

In addition to between individuals, social interaction often occurs between different social groups, and children are often faced with resource inequality between groups. According to the social reasoning developmental (SRD) perspective, in intergroup contexts, developing children not only show concerns about fairness but also weigh group concerns such as group identification, group norms, and group status in their reasoning about resource allocation (Rutland and Killen, 2017; Killen et al., 2018). Thus, resource allocation in intergroup contexts may be more complicated than that in interindividual contexts, and group concerns may have an effect on children’s resource allocation. For example, children’s ingroup preference or considerations for group status affect children’s distributive justice (Olson et al., 2011; Li et al., 2014; Paulus, 2016; Yu et al., 2016). Therefore, children may react differently to inequality when facing resource inequality between groups. Previous studies have reached inconsistent conclusions regarding how children balance fairness and group concerns in resource allocation in the context of intergroup inequality and whether they perpetuate or rectify preexisting inequality.

The system justification theory (SJT) suggests that people consider the existing status to be just and reasonable, and they are motivated to maintain their status (Jost and Banaji, 1994; Olson et al., 2011). Some results of previous studies in children are consistent with this theory. Children show a preference for high-status races and wealthy groups, believing that high-status children are more capable and popular (Hailey and Olson, 2013; Horwitz et al., 2014; Shutts et al., 2016). In intergroup resource allocations, when resources are unequal between certain groups (e.g., between Asian and White men or between two minimal groups), children allocate more resources to members of advantaged groups and perpetuate intergroup inequality (Olson et al., 2011; Elenbaas and Killen, 2016).

However, other studies suggest that children take the disadvantages of certain groups into account and rectify inequality (Rutland and Killen, 2017). For example, when resources are unequal between Black and White people, older school-aged children allocate more resources to disadvantaged group members to rectify resource inequality between racial groups, whereas younger preschool children perpetuate resource inequality (Olson et al., 2011; Elenbaas and Killen, 2016). This may be because in the process of resource allocation, older children can consider the difference in status between Black and White people based on their previous experience and thus pay more attention to equality between two groups with different statuses, while the difference in status between other groups (e.g., between Whites and Asians or two minimal groups) is not as obvious (Olson et al., 2011; Elenbaas and Killen, 2016).

It can be seen that children’s resource allocation behaviors in the context of intergroup inequality may be different depending on which social groups are involved. In reality, social inequality is often reflected between some specific social groups, such as different genders, ethnicities, or regional groups (Olson et al., 2011; Ridgeway, 2014; Normile, 2017). Disparities in group status between such groups often lead to the inequality of resource distributions. For example, resource inequality may emerge due to differences in regional economic status, such that people in some regions are richer and enjoy more and better resources than people in other regions (Normile, 2017). When intergroup inequality is caused by different factors, there may be differences in children’s intergroup resource allocation. Previous studies have shown that children consider the origins of inequality when faced with existing resource inequality between individuals. They recognize and accept resource inequality caused by impartial internal factors (e.g., performance and effort) and disapprove of and rectify inequality derived from structural factors (e.g., gender and race) (Shaw, 2016; Rizzo et al., 2020). The question is as follows: when intergroup resource inequality is based on internal and structural origins, will children of different ages rationalize and maintain inequality in resource allocations (Olson et al., 2011; Horwitz et al., 2014; Shutts et al., 2016) or will they show aversion to inequality and compensate resources for the disadvantaged groups? (Blake and McAuliffe, 2011; Olson et al., 2011; Elenbaas and Killen, 2016; Rizzo and Killen, 2016). In other words, the origins of intergroup resource inequality may play a role in children’s resource allocations between groups; this topic will be explored in this study.

Ingroup bias also has an effect on children’s distributive justice in intergroup contexts. Children allocate more resources to friends than to strangers or disliked peers (Fehr et al., 2008; Yu et al., 2016), and they also show preferences for ingroup members with respect to race, gender, or minimal groups in allocation (Renno and Shutts, 2015; Sparks et al., 2017). In some situations, young children can also fairly distribute resources between ingroup and outgroup members. For example, when the allocation method can be selected, young children choose to distribute fairly to ingroup and outgroup members by using a fair procedure (Li et al., 2019). When the merits of ingroup and outgroup members are different, young children can allocate equitably to ingroup and outgroup members based on their merits without showing a preference for the ingroup (Xiao et al., 2019). This may be due to the possibility that children’s preference for fairness is stronger than their ingroup preference when there is an obvious and impartial basis for distribution (e.g., fair procedures and merit).

When facing existing inequalities between ingroups and outgroups, young children are more likely to reject inequality that disadvantages the ingroups and allocate more to the disadvantaged ingroups; however, with age, children’s ingroup preferences weaken, and they tend to rectify the disadvantages of both ingroups and outgroups (Jordan et al., 2014; Elenbaas et al., 2016). This study further explores whether children’s ingroup bias in intergroup resource allocation varies due to the origins of the inequality between ingroups and outgroups.

In this study, performance was selected as an internal factor, region was selected as a structural factor, and intergroup resource inequalities based on differences in performance or region were presented. This is because in children’s daily education environments, academic performance is an important indicator for evaluating children. Schools with different academic performances may obtain unequal educational resources. Disparities in regional economic status (such as differences between urban and rural areas) can also lead to inequality in educational resources (Normile, 2017). Common school supplies were selected as resources to be allocated in these contexts.

Children aged 5–6 and 10–11 years were recruited. Previous studies have found that 5- to 6-year-old children can rectify inequality between individuals in resource allocation (Li et al., 2014), and children aged 10–11 can rectify intergroup inequality (Elenbaas and Killen, 2016; Elenbaas et al., 2016). Compared with children aged approximately 6 years old, 10-year-olds can better understand the reasons for inequality (Sigelman, 2012). Therefore, the ages between 5–6 and 10–11 years may be a critical stage in which children’s understanding of intergroup inequality and intergroup allocation behavior develops.

The role of the origins of intergroup resource inequality in the intergroup resource allocation of children aged 5–6 and 10–11 years was investigated in this study. There were three situations where intergroup resource inequality without an explicit origin, intergroup resource inequality based on an internal factor (levels of performance), or intergroup resource inequality based on a structural factor (regional advantages or disadvantages) were presented. We explored how children weigh group concerns and fairness principles to allocate resources and compared the differences in children’s resource allocation between different situations.

Based on previous research findings, we predicted that the origins of intergroup resource inequality affected children’s intergroup resource allocation. Compared with the situation of presenting inequality with no explicit origin, when intergroup resource inequality was based on an internal factor, children of both age groups would be more likely to perpetuate resource inequality, and when resource inequality was based on a structural factor, only older children (10- to 11-year-olds) would be more likely to rectify resource inequality (Olson et al., 2011; Elenbaas and Killen, 2016; Shaw, 2016; Rizzo et al., 2020).

## Experiment 1

The aim of experiment 1 was to explore children’s intergroup resource allocation when preexisting intergroup resources inequality had no explicit origin. To exclude the influence of past experience in real groups, minimal groups were used in this experiment. Referring to previous studies (Dunham et al., 2011; Olson et al., 2011), the ingroup and outgroup were differentiated according to the color of stickers that children received.

### Methods

#### Participants

Children aged 5–6 years old in kindergarten (*n* = 34, *M* = 6.00, SD = 0.30, range = 5.49–6.49, and 50% girls) and children aged 10–11 years old in fifth grade (*n* = 34, *M* = 11.21, SD = 0.26, range = 10.73–11.62, and 50% girls) participated in this experiment. The sample size of each age group in the experiment was larger than the minimum sample size (*n* = 17) required to detect a medium effect size (*f* = 0.25) with 80% power based on G^∗^Power 3.1. Children were recruited from an ordinary kindergarten and primary school in Beijing. According to the information provided by the teachers and the community where the kindergarten and the school were located, most children’s families were from middle-class background. Informed consent was obtained from caregivers and children themselves. This study was approved by the ethics committee of the Institute of Psychology, Chinese Academy of Sciences.

#### Procedure

The experiment was conducted in a quiet reading room, and children were tested individually by trained research assistants. The research assistants described the intergroup situations using pictures. There were two intergroup conditions: one in which the ingroup was at a resource advantage (i.e., outgroup at a disadvantage) and the other in which the ingroup was at a resource disadvantage (i.e., outgroup at an advantage). Each participant completed both conditions, and the order of the two scenarios was balanced. After each scenario was presented, children completed the resource allocation task. The entire experiment lasted approximately 15 min.

Before the experiment started, children were randomly assigned a sticker with different colors (half received red stickers and half received green stickers). In the ingroup advantaged scenario, participants were presented with pictures of two groups and told that they were in the same group as children with the same color stickers. The gender of characters in intergroup situations always matched that of children. Next, children were told how many pens the two groups had: “Now the students have some pens for learning. The first student in your green/red star group has four pens, and the student in the red/green star group has one pen. Look at others. The second student in the green/red star group also has four pens, and the second student in the red/green star group also has one pen.” In the ingroup disadvantaged scenario, the children were told how many books the ingroup and another outgroup (i.e., blue star group) had: each student in the ingroup had one book and each student in the outgroup had four books.

After each scenario was presented, the experimenter asked participants two questions to ensure the establishment of participants’ group membership and participants’ knowledge of the intergroup contexts, “Which students are you in/not in a group with?” and “Which group of students have more pens/books?” All participants in this study answered the questions correctly and proceeded to the following resource allocation phase. Then, participants were asked to allocate pens/books to students in the two groups who did not have pens/books: “Now there is another student in each group who does not have pens/books yet. Here are five pens/books. You can distribute them to the two students. You can choose to distribute all five pens/books or return some to me if you do not want to distribute all of them. Now, please start to put the pens/books in the corresponding envelopes (showing two envelopes with pictures of each student).” After the allocations were completed, children were asked to explain the reasons for their choices.

#### Data Analysis Plan and Coding

First, this experiment used mixed analysis of variance (ANOVA) to explore the differences in the number of resources children allocated to each group. Children’s allocations between the two groups were categorized into three types: rectifying inequality (allocating more to students in the disadvantaged group), perpetuating inequality (allocating more to students in the advantaged group), and equal distribution. The chi-square test of independence was used to analyze the differences in children’s resource allocation patterns. Finally, to understand the motivations of children of different ages and their recognition of the intergroup allocation contexts, children’s justifications for their allocation behavior were collected, coded, and analyzed using chi-square tests.

Based on previous studies (Elenbaas and Killen, 2016; Elenbaas et al., 2016), children’s justifications for allocations were divided into four categories, “Equality,” “Preference for the advantaged,” “Compensation for the disadvantaged,” and “Other.” Examples for “Equality” justifications include “I want to distribute equally” and “Everyone should be treated equally.” In the “Preference for the advantaged” category, example statements include “It should be the same in the same group” and “higher rank, higher treatment.” Examples of the “Compensation for the disadvantaged” category include “everyone needs books, maybe they are poor, so give them one more.” Justifications that cannot be categorized into the above three categories, such as “I want it like this” and “I don’t know” were coded as “Other.” Two coders blind to the purpose of the study were invited to independently encode 25% of the data (*n* = 36). The interrater reliability between the coders was computed, Cohen’s *k* = 0.96. The remaining 75% of the data were coded by the two coders (each one completed half of the cases).

### Results

#### Children’s Resource Allocation

**FIGURE 1:**
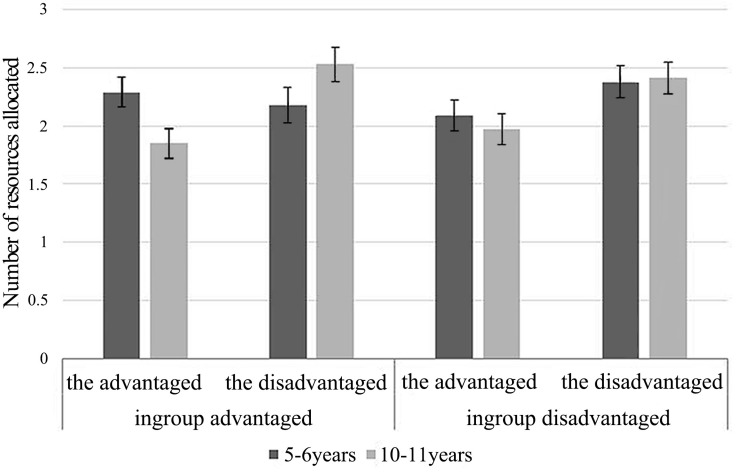
Number of resources children allocated between groups in experiment 1.

Data analyses were performed by SPSS 20.0. A 2 (age: 5–6 and 10–11) × 2 (ingroup status: ingroup advantaged and ingroup disadvantaged) × 2 (recipients: the advantaged and the disadvantaged) three-factor mixed-design ANOVA with the number of school supplies allocated to recipients as the dependent variable was conducted. The results are shown in Figure 1. There was a statistically significant three-way interaction among age, ingroup status, and recipients, *F*(1, 66) = 8.05, *p* = 0.006, and η^2^*_p_* = 0.11.

To further analyze the three-way interaction, two 2 (age) × 2 (recipients) ANOVAs were conducted. When the ingroup was at an advantage, a significant interactive effect was found, *F*(1, 66) = 4.69, *p* = 0.034, and η^2^*_p_* = 0.07. Further simple effect tests revealed that older children allocated more to the disadvantaged group member (*M* = 2.53 and SD = 0.15) than the advantaged group member (*M* = 1.85 and SD = 0.13), *p* = 0.011, whereas there was no significant difference in younger children’s allocations (*M* = 2.18, SD = 0.15; *M* = 2.29, SD = 0.13), *p* = 0.65. Moreover, younger children allocated more resources to advantaged group members than older children, *p* = 0.018, while no significant age difference was found in children’s allocation to the disadvantaged group member, *p* = 0.097. When the ingroup was at a disadvantage, no significant interactive effect was found, so children of different ages did not show significant differences in resource allocation to the advantaged (older children: *M* = 1.97 and SD = 0.13; younger children: *M* = 2.09 and SD = 0.13) or disadvantaged group members (older children: *M* = 2.41 and SD = 0.14; younger children: *M* = 2.38 and SD = 0.14), *p*_s_ > 0.05.

In addition, for younger children, a significant two-way interaction was found by a 2 (ingroup status) × 2 (recipients) ANOVA, *F*(1, 33) = 8.56, *p* = 0.006, and η^2^*_p_* = 0.21. A simple effect test showed that, compared with the situation in which the ingroup was advantaged, younger children allocated more resources to the disadvantaged ingroup members and less to the advantaged outgroup members when the ingroup was resource disadvantaged, *p* = 0.017, and older children’s resource allocation in the two conditions was not significantly different, *p_s_* > 0.05.

**FIGURE 2:**
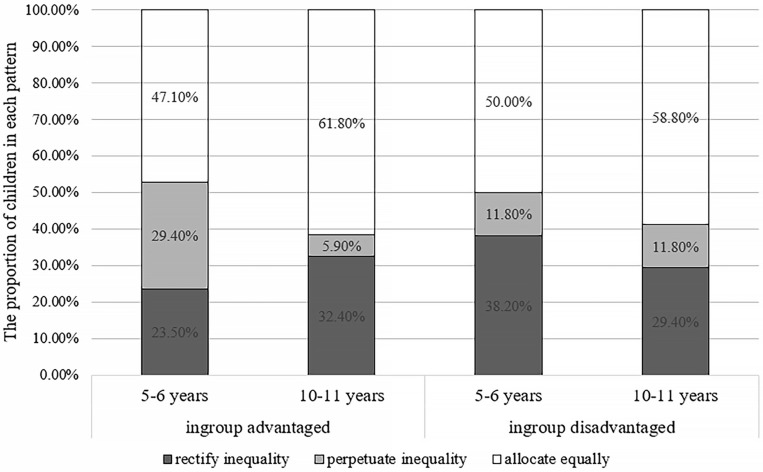
Percentage of allocation patterns between groups in experiment 1.

The analysis of the children’s resource allocation patterns showed that both younger and older children mainly distributed resources in an equal way. When the ingroup was at an advantage, the chi-square test of independence indicated that there were differences in allocation patterns between younger children and older children, χ^2^(2, *N* = 68) = 6.46, *p* = 0.042, Cramer’s *V* = 0.31, and *z*-tests indicated that younger children were more likely to perpetuate resource inequality than older children, *p* < 0.05. No significant age difference existed in other allocation patterns, *p_s_* > 0.05. When the ingroup was at a disadvantage, there was no significant difference in the allocation patterns between children of different ages, *p* = 0.79, as shown in Figure 2.

The above results showed that younger children were more likely to rectify resource inequality and less likely to perpetuate inequality when their ingroup was at a disadvantage than when their outgroup was disadvantaged, thus showing ingroup preference. Moreover, older children were more likely to rectify inequality or allocate equally regardless of whether their ingroup was at an advantage or disadvantage. However, in terms of the allocation patterns, most children of different ages make equal allocation irrespective of the conditions.

#### Justifications for Resource Allocation

The chi-square test of independence showed that regardless of whether the ingroup was at a resource advantage or a disadvantage, there was no significant difference in the justifications for their allocation behavior between age group, χ^2^(3, *N* = 68) = 4.20, *p* = 0.24; χ^2^(3, *N* = 68) = 3.10, *p* = 0.38. Both younger and older children paid more attention to the equality of resources and mainly mentioned in their explanations that resources should be divided equally. The proportion of each category of children’s justifications for their own allocation behavior is shown in Table 1.

**TABLE 1:** Children’s justifications for allocation in the context of inequality without an explicit origin.

	5–6 years old		10–11 years old	
		
	Ingroup advantaged (%)	Ingroup disadvantaged (%)	Ingroup advantaged (%)	Ingroup disadvantaged (%)
Preference for the advantaged	8.80	5.90	2.90	2.90
Compensation for the disadvantaged	20.60	17.60	26.50	26.50
Equality	47.10	50.00	61.80	58.80
Other	23.50	26.50	8.80	11.80

### Discussion

This experiment explored how children distributed school supplies between ingroup and outgroup members when witnessing a preexisting intergroup school supplies inequality. We found that both younger and older children tended to allocate resources in an equal manner when presented with preexisting intergroup resource inequality with no explicit origin. Compared with the situation where the outgroup was disadvantaged, more younger children rectified resource inequality when their ingroup was at a disadvantage, showing ingroup preference.

Previous studies have also found ingroup preferences in younger children when resources are unequal. Children aged 5–6 years show their preference for friends in resource allocation, even when friends are richer than strangers (Paulus, 2016), and they are more likely to reject and rectify the disadvantages of ingroups (Jordan et al., 2014; Elenbaas et al., 2016). Nevertheless, as children’s ingroup preference decreases with age, older children are more likely to rectify the disadvantages of both ingroup and outgroup members due to fairness preference (Jordan et al., 2014; Elenbaas et al., 2016). It is possible that younger children have a stronger motivation for ingroup preference in resource allocation than older children, whereas older children’s motivation for maintaining fairness is stronger than their ingroup preference, thus making them more likely to allocate resources equally or rectify inequality. Notably, in this experiment, younger and older children mainly distributed equally when faced with unequal resources between groups. The difference is that previous studies have shown that Western children aged 3.5–11.5 years perpetuate resource inequality between two minimal groups (Olson et al., 2011). This may be because Chinese children pay more attention to the harmony of social relations than Western children and tend to allocate resources equally (Chai and He, 2017; Li et al., 2019). In addition to intergroup resource inequality without an explicit origin, intergroup inequality based on certain origins may have different effects on children’s intergroup resource allocation (Olson et al., 2011; Shaw, 2016; Rizzo et al., 2020), which was examined in experiment 2.

## Experiment 2

Experiment 2 investigated the resource allocation of children in intergroup contexts when the preexisting resource inequality between groups was based on a structural factor (i.e., region) or an internal factor (i.e., performance).

### Methods

#### Participants

Children aged 5–6 years old in kindergarten (*n* = 34, *M* = 6.08, SD = 0.39, range = 5.48–6.64, and 50% girls) and children aged 10–11 years old in fifth grade (*n* = 34, *M* = 11.31, SD = 0.32, range = 10.79–11.99, and 50% girls) participated in this experiment. The rest of the information on the participants was the same as that in experiment 1.

#### Procedure

Except for the intergroup situations presented, the experimental procedures and tasks were the same as those in experiment 1. All participants witnessed intergroup resource inequality situations based on intergroup differences in region and performance, and the order in which the situations were presented was balanced among the participants.

##### Intergroup Contexts Based on Region

In the ingroup-advantaged scenario, pictures of houses and schools/kindergartens of two groups were presented, including one group living in the same place as the participants (Beijing) and one group living in a different place (Li Village; a fictional place was used to avoid the influence of participants’ experience). The number of pens owned by students in the two places was then described: each student from Beijing had four pens, and each student from Li Village had one pen. Then, the reason for the difference in the number of pens between the two groups was explained (i.e., students’ families in Beijing were richer than students’ families in Li Village, and their housing and schools/kindergartens were in better conditions in Beijing; thus, they had more school supplies than students in Li Village).

In the ingroup-disadvantaged scenario, students of another group from Shangzhou (also a fictional place) were introduced to participants, and pictures of two groups in Beijing and Shangzhou were presented. Next, the number of stationery sets in each group was described, and the reason for the difference in the number of stationery sets between the two groups was also described (i.e., students in Shangzhou were richer than students in Beijing, and their housing and schools/kindergartens were in better conditions).

##### Intergroup Contexts Based on Performance

Participants first participated in the “I know” game so that they could identify the group to which they belonged in the intergroup situation based on performance. To involve participants in the ingroup-advantaged and ingroup-disadvantaged scenarios, we ensured that they answered three out of five questions correctly within the prescribed time by presenting five questions of different difficulty levels according to the grade of children. At the end of the game, each participant was rewarded with a red star sticker.

In the ingroup-advantaged scenario, students in the red star group (the ingroup) and students in the gray star group (the outgroup) were introduced, and the number of pens they owned was described. Then, the reason for the different numbers of pens between the in- and outgroups was explained: each student in the red star group answered three questions correctly, and no students in the gray star group answered a question correctly, and thus, students in the red star group had four pens each, whereas students in the gray star group had one pen each.

In the ingroup-disadvantaged scenario, students of another group (multicolored star group) were presented, and the number of books owned by the ingroup (the red star group) and the outgroup (the multicolored star group) was shown. Children were told that the multicolored star group members received four books each, and the red star group members received one book each because the multicolored star group members answered all five questions correctly and performed better.

#### Data Analysis Plan and Coding

Differences in children’s resource allocation behavior and justifications when intergroup resource inequalities were based on region and performance were analyzed. The results of this experiment were compared with the results of experiment 1 to explore the role of the origins of intergroup resource inequality in children’s resource allocation.

Children’s reasons for allocation were divided into the same four categories as in experiment 1. The “Preference for the advantaged” category involved explanations such as “Beijing is richer” and “They answered more, so I praise them.” In the category of “Compensation for the disadvantaged,” children mentioned explanations such as “Li Village is very poor and can’t afford it” and “Their group has too few and should have more.” The other two categories of “Equality” and “Other” were the same as those in experiment 1. After two independent coders coded 25% of the data (*n* = 72), the interrater reliability was calculated, Cohen’s *k* = 0.84.

### Results

#### Children’s Resource Allocation

A 2 (origins of inequality: region, performance) × 2 (age: 5–6 and 10–11) × 2 (ingroup status: ingroup advantaged and ingroup disadvantaged) × 2 (recipients: the advantaged and the disadvantaged) four-way mixed-design ANOVA with the number of school supplies allocated as the dependent variable was conducted. The results showed that there was a significant four-way interaction, *F*(1, 66) = 12.17, *p* = 0.001, and η^2^*_p_* = 0.16. Follow-up tests were performed with two 2 (age) × 2 (ingroup status) × 2 (recipients) three-way ANOVAs.

**FIGURE 3:**
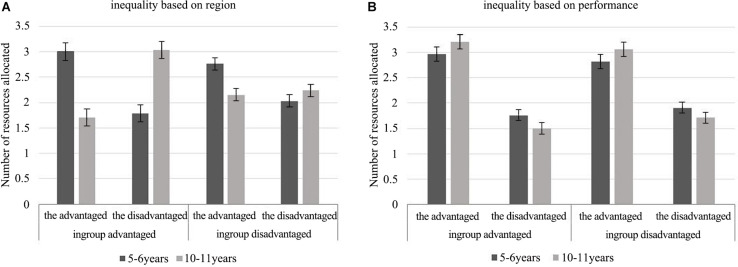
Number of resources children allocated between groups in the context of inequality based on region **(A)** or based on performance **(B)** in experiment 2.

For intergroup resource inequality based on region, the three-way interaction was significant, *F*(1,66) = 16.12, *p* < 0.001, η^2^*_p_* = 0.20, and further analyses showed that the age × recipients interaction was significant, regardless of whether the ingroup was at an advantage or at a disadvantage, *F*(1,66) = 30.81, *p* < 0.001, η^2^*_p_* = 0.32; *F*(1,66) = 6.71, *p* = 0.012, η^2^*_p_* = 0.09. Simple effects analysis indicated that when intergroup inequality was based on region, younger children allocated more school supplies to members of the advantaged group than to members of the disadvantaged group in both the ingroup-advantaged (*M* = 3.00, SD = 0.17 versus *M* = 1.79, SD = 0.17, and *p* < 0.001) and ingroup-disadvantaged (*M* = 2.77, SD = 0.12 versus *M* = 2.03, SD = 0.12, and *p* = 0.002) conditions. When older children were in an advantaged group, they allocated more to the disadvantaged group members (*M* = 3.03 and SD = 0.17) than the advantaged ingroup members (*M* = 1.71 and SD = 0.17), *p* < 0.001; moreover, compared with younger children, older children allocated less to the advantaged ingroup members, *p* < 0.001, and more to the disadvantaged group members, *p* < 0.001. When older children were in a disadvantaged group, no significant difference was found in their resource allocations between the advantaged and disadvantaged groups (*M* = 2.15 and SD = 0.12; *M* = 2.24 and SD = 0.12), *p* = 0.70, but they still allocated fewer resources to members of the advantaged group than younger children, *p* = 0.001. These results are shown in Figure 3.

**FIGURE 4:**
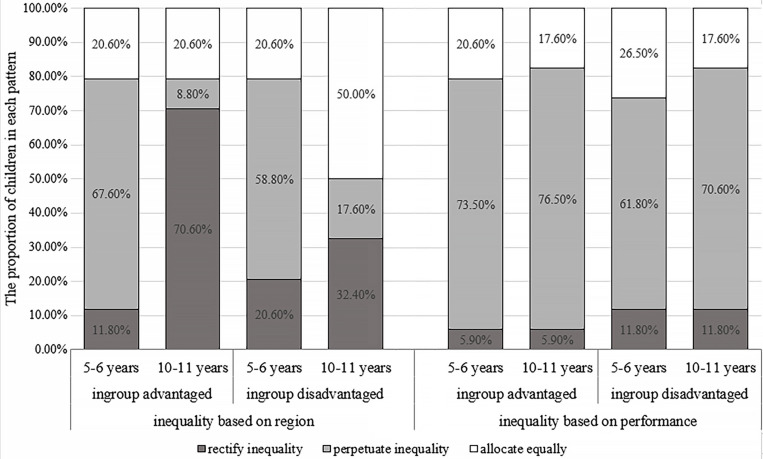
Percentage of allocation patterns between groups in experiment 2.

The analysis results of children’s allocation patterns when intergroup inequality was based on region were consistent with the above results, as shown in Figure 4. Younger children were significantly different from older children in the allocation patterns, regardless of whether the ingroup was at an advantage or a disadvantage, χ^2^(2, *N* = 68) = 31.81, *p* < 0.001, Cramer’s *V* = 0.66; χ^2^(2, *N* = 68) = 12.64, *p* = 0.002, Cramer’s *V* = 0.43. More older children chose to rectify resource inequality in the ingroup-advantaged condition or allocate equally in the ingroup-disadvantaged condition than young children, *p*_s_ < 0.05; young children were more likely to perpetuate resource inequality than older children, *p*_s_ < 0.05.

When intergroup resource inequality was based on performance, the 2 (age) × 2 (ingroup status) × 2 (recipients) follow-up ANOVA showed that the ingroup status × recipients two-way interaction was significant, *F*(1, 66) = 5.67, *p* = 0.02, and η^2^*_p_* = 0.08. Both older and younger children allocated more to members of the advantaged group than to members of the disadvantaged group, regardless of whether their ingroup was at an advantage (older children: *M* = 3.21, SD = 0.14 versus *M* = 1.50, and SD = 0.11; younger children: *M* = 2.97, SD = 0.14 versus *M* = 1.77, and SD = 0.11) or at a disadvantage (older children: *M* = 3.06, SD = 0.14 versus *M* = 1.71, and SD = 0.11; younger children: *M* = 2.82, SD = 0.14 versus *M* = 1.91, and SD = 0.11), *p_s_* < 0.001, as shown in Figure 3. There was no significant difference between the distribution of older and younger children, *p_s_* > 0.05. The analysis of children’s allocation patterns yielded similar results, there was also no significant age difference, *p*_s_ > 0.05, as illustrated in Figure 4.

In summary, in the context of intergroup inequality based on region, younger children allocated more to the advantaged group, whereas older children allocated more to the disadvantaged group or in an equal way. When the intergroup resource inequality was based on performance, all children distributed more to the advantaged group members.

#### Justifications for Resource Allocation

The proportions of the four categories of children’s justifications for their resource allocation are also shown in Table 2. The chi-square test of independence showed that when resource inequality was based on region, the differences in children’s interpretations of allocation behaviors were significant, regardless of whether the ingroup was at an advantage or a disadvantage, χ*^2^*(3, *N* = 68) = 34.08, *p* < 0.001, and Cramer’s *V* = 0.71; χ*^2^*(3, *N* = 68) = 14.46, *p* = 0.002, and Cramer’s *V* = 0.46. Younger children were more likely to mention a preference for advantaged groups than older children, *p_s_* < 0.05, whereas older children were more likely to mention compensation for the disadvantaged or equality than younger children, *p_s_* < 0.05. When intergroup resource inequality was based on differences in performance, there was no significant difference in the explanations of allocation behaviors between children of different ages, regardless of whether their ingroup was advantaged or disadvantaged, χ*^2^*(3, *N* = 68) = 5.73, *p* = 0.11; χ*^2^*(3, *N* = 68) = 7.18, *p* = 0.06. Younger children and older children both mentioned their preferences for the advantaged.

**TABLE 2:** Children’s justifications for allocation in the context of inequality with different origins.

	Inequality based on region				Inequality based on performance			
		
	5–6 years old		10–11 years old		5–6 years old		10–11 years old	
				
	Ingroup advantaged (%)	Ingroup disadvantaged (%)	Ingroup advantaged (%)	Ingroup disadvantaged (%)	Ingroup advantaged (%)	Ingroup disadvantaged (%)	Ingroup advantaged (%)	Ingroup disadvantaged (%)
Preference for the advantaged	52.90	47.10	8.80	11.80	55.90	50.00	73.50	58.80
Compensation for the disadvantaged	8.80	17.60	73.50	29.40	2.90	8.80	5.90	23.50
Equality	20.60	17.60	17.60	50.00	20.60	20.60	17.60	14.70
Other	17.60	17.60	0.00	8.80	20.60	20.60	2.90	2.90

#### Role of Origins of Inequality

To explore the impact of resource inequality with a structural origin on children’s intergroup resource allocation, a 2 (origins of inequality: region and no origin) × 2 (age: 5–6 and 10–11) × 2 (ingroup status: ingroup advantaged and ingroup disadvantaged) × 2 (recipients: the advantaged and the disadvantaged) four-factor mixed ANOVA was conducted. The results showed that the interaction effect of the four factors was significant, *F*(1, 132) = 4.82, *p* = 0.03, and η^2^*_p_* = 0.04. Two 2 (age) × 2 (origins of inequality) × 2 (recipients) three-way ANOVAs were conducted to further explore the four-way interaction. When the ingroup was at an advantage, the three-way interaction was significant, *F*(1, 132) = 8.80, *p* = 0.004, η^2^*_p_* = 0.06, and age × origins of inequality interactions were significant when allocating to the advantaged or the disadvantaged, *F*(1, 132) = 8.30, *p* = 0.005, η^2^*_p_* = 0.06; *F*(1, 132) = 7.80, *p* = 0.006, η^2^*_p_* = 0.06, while the origins of inequality × recipients interaction was found when the ingroup was at a disadvantage, *F*(1, 132) = 8.35, *p* = 0.005, η^2^*_p_* = 0.06.

Simple effects tests showed that younger children allocated more to the advantaged group members when intergroup resource inequality was based on the region than when inequality had no explicit origin, regardless of whether their ingroup was advantaged or disadvantaged, *p* = 0.001 and *p* < 0.001. Older children whose ingroup was in the advantaged position allocated more resources to the disadvantaged group members when intergroup inequality was based on the region than when there was no origin of inequality, *p* = 0.027. No significant difference existed in other conditions, *p_s_* > 0.05.

Similarly, the impact of resource inequality with an internal origin on children’s resource allocation was explored. The results of four-way mixed ANOVA showed that the four-way interaction effect was significant, *F*(1, 132) = 3.96, *p* = 0.049, and η^2^*_p_* = 0.03. Follow-up tests showed that when the ingroup was at an advantage, the origins of inequality × age × recipients three-way interaction was found, *F*(1, 132) = 6.92, *p* = 0.01, η^2^*_p_* = 0.05, and two age × origins of inequality interactions were also significant when allocating to the advantaged or the disadvantaged, *F*(1, 132) = 6.27, *p* = 0.013, and η^2^*_p_* = 0.05; *F*(1, 132) = 5.78, *p* = 0.018, and η^2^*_p_* = 0.04. Both younger and older children significantly allocated more to the advantaged group members (*p* = 0.001 and *p* < 0.001) and less to the disadvantaged group members (*p* = 0.025 and *p* < 0.001) when intergroup resource inequality was based on performance than when there was no explicit origin of inequality. When the ingroup was at a disadvantage, the origins of inequality × recipients interaction was also significant, *F*(1, 132) = 37.97, *p* < 0.001, and η^2^*_p_* = 0.22. Simple effects tests showed the same results as when the ingroup was at an advantage, *p_s_* < 0.01.

### Discussion

This experiment explored the role of different origins of intergroup resource inequality in children’s intergroup resource allocation. Younger children tended to perpetuate resource inequalities by allocating more resources to group members who were already advantaged regardless of whether the inequality was due to region or performance differences. Older children paid more attention to the needs, performance, and equality of group members, and thus, they were more inclined to rectify resource inequality or allocate resources equally when intergroup resource inequality with the regional origin and perpetuated resource inequality based on performance.

In previous Western studies, when intergroup resource inequality is based on structural factors (e.g., Black and White groups), older children always allocate more resources to groups with fewer resources, while younger children do not (Olson et al., 2011; Elenbaas and Killen, 2016; Elenbaas et al., 2016). These findings are consistent with the results of the current study suggesting that older children generally rectify inequality based on structural factors. However, this study further shows that older children can differentiate intergroup resource inequalities based on internal and structural factors. When facing resource inequality based on an internal factor (e.g., performance), children were more likely to perpetuate the inequality.

We also noticed that older children tended to rectify inequality when they were at a regional advantage, while allocated equally when they were at a regional disadvantage. The economic development gap between urban and rural areas is huge in China, and in some poor regions, children’s living and learning environments are far worse. We believe that older children are aware of this gap (Kuang et al., 2020), so most older children rectified the inequality when their ingroup was at an advantage to achieve structural equality. When their ingroup was at a disadvantage, older children mentioned that there were enough learning resources in both regions (as shown in the experimental scenario), and hence, they allocated resources equally to achieve distributive equality instead of fighting for structural equality for themselves. One possibility is that Chinese children are not very assertive and the cultural encouragement of other-regarding preference and group harmony makes it easier for them to fight for others rather than fighting for themselves for structural equality. Therefore, even though they challenged the preexisting inequality between regions in both conditions, when in advantaged groups, they rectified inequality by allocating more to outgroup members; however, when in disadvantaged groups, they tended to allocate resources equally instead of allocating more to themselves to rectify the regional inequality.

In addition, by comparing with experiment 1, we find that origins of intergroup resource inequality (based on either an internal or structural factor) have an important influence on children’s resource allocation behaviors. Children mainly distributed resources equally, and younger children showed ingroup preference when there was no explicit origin of intergroup inequality. However, in the context of intergroup inequalities with origins, younger children were more likely to perpetuate inequalities, whereas older children were more likely to rectify inequality due to region and perpetuate inequality due to performance, and they did not show ingroup preference. This may be because children tend to allocate resources equally to maintain harmony when there is no obvious clue to be used in resource allocation, and ingroup preference becomes a motivation for younger children’s allocation. Nevertheless, children of different ages are both more likely to make allocations based on different clues when there are some explicit bases for allocation (Li et al., 2019; Xiao et al., 2019).

## General Discussion

This study explored the role of the origins of resource inequality in children’s intergroup resource allocation in the context of preexisting intergroup resource inequality. We found that when inequality was not based on an explicit origin, children mainly distributed resources equally, and younger children manifested ingroup favoritism. When different origins of intergroup resource inequality were introduced, children were more likely to recognize inequality caused by an internal factor, and older children would further rectify inequality based on a structural factor. Based on the SRD perspective, this study extends previous studies and aims to explain how children weigh fairness and group concerns in the context of intergroup resource inequality with different origins (Rutland and Killen, 2017; Killen et al., 2018).

When facing intergroup resource inequality with no explicit origin, Chinese children may be more concerned about equality in the amount of resources allocated (Chai and He, 2017; Li et al., 2019). Although younger children have a motivation for ingroup favoritism (Renno and Shutts, 2015; Yu et al., 2016), motivation for equality is stronger than their considerations about group factors for most children. When origins of intergroup resource inequality are salient, children’s attention to group factors might be strengthened, and they are thus more likely to show a tendency to perpetuate or rectify the existing intergroup inequalities due to group characteristics.

Children aged 5–6 years old maintained the existing resource inequalities, displaying a tendency of using system justification for intergroup resource inequalities with clear origins (Jost and Banaji, 1994; Olson et al., 2011). This tendency may occur because when witnessing status differences between groups, younger children have a stronger preference for high-status group members and a more positive evaluation of them (Horwitz et al., 2014; Shutts et al., 2016). They may believe that the existing inequalities between the high- and low-status groups are reasonable and thus perpetuate the advantages of high-status groups in resource allocation (Olson et al., 2011; Shutts et al., 2016). With increasing age, children do not always maintain intergroup resource inequalities, and they can gradually distinguish intergroup resource inequalities based on different origins. Older children maintained intergroup resource inequality due to differences in performance but rectified intergroup inequality caused by regional differences. These results are similar to previous research findings indicating that when older children and adolescents consider the system to be justified, they attribute the existing inequalities to internal reasons such as ability and effort rather than structural reasons such as job and educational opportunities (Sigelman, 2012; Godfrey et al., 2019). It is possible that older children have a more complete understanding of fairness. As suggested by the impartiality account of fairness, they recognize that inequality caused by impartial factors such as performance, needs, and random procedures is fair, while inequality based on factors such as race and gender is partial (Shaw, 2016). Therefore, when resource inequality between groups is based on an impartial origin (i.e., internal performance), older children allocate resources according to performance and perpetuate intergroup resource inequality. However, when intergroup inequality is brought about by a partial factor (i.e., region), they may pay more attention to the equality and needs of the disadvantaged groups and therefore are willing to rectify the existing inequality of the system.

We found that older children aged 10–11 years were more likely to recognize intergroup resource inequality based on an internal factor and to rectify the intergroup inequality based on a structural factor. This is different from previous research findings on resource inequality between individuals indicating that children aged 3–8 years old can distinguish resource inequality based on internal or structural factors in resource allocation (Rizzo et al., 2020). This suggests that in early childhood, children develop a certain understanding of the different factors that lead to resource inequality, but compared to interindividual inequality, children may develop sensitivity to different origins of intergroup resource inequality later in childhood, and only older children rectify resource inequality based on a structural factor. This may be because children have a stronger tendency toward system justification in intergroup contexts, and they are more inclined to rationalize the intergroup differences in status and resources, thereby perpetuating the system inequality (Olson et al., 2011). As they grow older, children also gain a deeper understanding of social inequality based on different origins, and accordingly, only older children can rectify inequality based on unreasonable origins (Olson et al., 2011; Elenbaas and Killen, 2016).

On the basis of previous studies on intergroup resource inequality, this study distinguished the impact of different origins of intergroup resource inequality on children’s resource allocation and shows that origins of intergroup resource inequality play an important role in children’s intergroup allocation. There are some limitations in this study. First, this study was to explore children’s responses to intergroup inequality, and children were required to allocate resources to members of a group. Though children did consider group characteristics in their justification for allocation, we cannot completely rule out the possibility that in the process of resource allocation, some children may consider both group and individual characteristics. Similarly, when intergroup inequality was based on a structural factor, we made it clear to children that the reason for inequality is due to differences in economic levels between regions. They mostly mentioned the regions and their rich or poor conditions as a whole from children’s justification (for example, “Beijing is richer” and “there are fewer resources in Li Village”). We believe that children do consider structural factors, but we cannot rule out the possibility that they may also consider other related factors simultaneously. Future research can examine whether children can understand inequality based on structural factors more specifically. Second, in this study, only two age groups of children (5–6 and 10–11 years old) were recruited, and the development trend of children’s intergroup allocation behavior could not be examined. Future studies can track the developmental trajectories of children’s intergroup allocation in the same context using a longitudinal design. Third, this study used minimal groups to control the influence of children’s past interactive experiences, which might have caused children’s lower levels of identification with the ingroup. Future studies can examine actual social groups. Finally, participants in this study were all recruited from the same kindergarten or elementary school in Beijing, and thus, their socioeconomic status and development experiences were relatively similar. Future research can recruit children from different socioeconomic backgrounds to obtain a more comprehensive understanding of children’s recognition of intergroup inequality in different social environments.

In summary, the study shows that origins of intergroup resource inequality affect children’s resource allocation. Younger children (5–6 years old) perpetuated intergroup resource inequality when it was based on internal or structural factors. Older children (10–11 years old) can distinguish different origins of intergroup resource inequality. They perpetuated intergroup inequality when it was based on an internal factor but they were more likely to rectify inequality that was based on a structural factor. Understanding children’s recognition of preexisting intergroup resource inequality and its origins may have important implications for education programs that aim to reduce social inequality and promote social justice.

## Data Availability Statement

The raw data supporting the conclusions of this article will be made available by the authors, without undue reservation.

## Ethics Statement

The studies involving human participants were reviewed and approved by the ethics committee of the Institute of Psychology, Chinese Academy of Sciences. Written informed consent to participate in this study was provided by the participants’ legal guardian/next of kin.

## Author Contributions

JA and LZ designed the experiments. JA collected and analyzed the data. JA, JY, and LZ wrote the manuscript. All authors contributed to the article and approved the submitted version.

## Conflict of Interest

The authors declare that the research was conducted in the absence of any commercial or financial relationships that could be construed as a potential conflict of interest.
